# Evaluation of 3D Vulnerable Objects’ Detection Using a Multi-Sensors System for Autonomous Vehicles

**DOI:** 10.3390/s22041663

**Published:** 2022-02-21

**Authors:** Esraa Khatab, Ahmed Onsy, Ahmed Abouelfarag

**Affiliations:** 1 Arab Academy for Science, Technology and Maritime Transport, Alexandria 1029, Egypt; abouelfarag@aast.edu; 2 School of Engineering, University of Central Lancashire, Preston PR1 2HE, UK; Aonsy@uclan.ac.uk

**Keywords:** autonomous driving, multiple object detection, 2D LiDAR, sensor fusion

## Abstract

One of the primary tasks undertaken by autonomous vehicles (AVs) is object detection, which comes ahead of object tracking, trajectory estimation, and collision avoidance. Vulnerable road objects (e.g., pedestrians, cyclists, etc.) pose a greater challenge to the reliability of object detection operations due to their continuously changing behavior. The majority of commercially available AVs, and research into them, depends on employing expensive sensors. However, this hinders the development of further research on the operations of AVs. In this paper, therefore, we focus on the use of a lower-cost single-beam LiDAR in addition to a monocular camera to achieve multiple 3D vulnerable object detection in real driving scenarios, all the while maintaining real-time performance. This research also addresses the problems faced during object detection, such as the complex interaction between objects where occlusion and truncation occur, and the dynamic changes in the perspective and scale of bounding boxes. The video-processing module works upon a deep-learning detector (YOLOv3), while the LiDAR measurements are pre-processed and grouped into clusters. The output of the proposed system is objects classification and localization by having bounding boxes accompanied by a third depth dimension acquired by the LiDAR. Real-time tests show that the system can efficiently detect the 3D location of vulnerable objects in real-time scenarios.

## 1. Introduction

Autonomous vehicles (AVs) have been considered a major research subject in recent years due to their multiple benefits. The average driver in England spends 235 h driving every year [1]; therefore, AVs offer passengers extra free time during their journeys. They also offer mobility to those who cannot drive, they reduce emissions and congestion, and they have the potential to enhance road safety [1,2,3,4]. Since the early 1990s, AVs have been the focus of attention in many research fields. Thus, several highly automated driver assistance capabilities have reached mass production.

In addition to the advantages of AVs, there are some challenges facing their widespread use, such as: legal terms, cybersecurity, traffic management strategies, moral and ethical challenges, and operational challenges [5,6].

The National Highway reports that 76% of all accidents are based solely on human error, while 94% involve human error [1]. Furthermore, in 2019, 25,080 motor vehicle fatalities were recorded by the Department of Transport in the United Kingdom [6]. The autonomous driving operation can be summarized in the following steps [7,8,9]:

- Self-localization.
- Environment recognition.
- Motion prediction.
- Decision Making.
- Trajectory generation.
- Ego-vehicle control.

### 1.1. Automation Levels 

Due to the differences in terminology used to describe autonomous driving, the Society of Automotive Engineers (SAE) has established a ranking for autonomous driving [10], which ranges from Level 0 (no automation) to Level 5 (automation under any Operational Design Domain). Until now, the market has not yet witnessed Level 5 AVs; however, there are concept cars, such as the Mercedes Benz S-Class, the VW Sedric, the Rinspeed Snap, etc., which are expected to be available by 2030. Ahangar et al. have explored the technical evolution in autonomous cars in [6].

### 1.2. Autonomous Vehicles’ Sensory Systems

Environment perception is achieved using the appropriate exteroceptive sensory system. Examples of exteroceptive sensors include: monocular and stereo-cameras, short- and long-range RADARs, ultrasonic sensors, and LiDARs, which is short for Light Detection and Ranging. 

Many survey papers have discussed different AVs’ sensory technologies [5,6,7,8,11,12,13,14,15,16,17,18,19]. In this paper, the proposed approach is based on performing sensor fusion between a 2D LiDAR and a monocular camera to achieve real-time object detection, classification, and 3D localization of objects in complex scenarios where objects are interacting and overlapping.

### 1.3. Related Work

In this subsection, we discuss the efficacy of images and point clouds when used in isolation and when integrated together in AVs in order to achieve object detection.

#### 1.3.1. Images Acquired by Cameras

Cameras are considered the primary vision sensor used for object detection for two reasons: they are one of the cheapest sensors that can be used on AVs, and they can acquire rich texture information. However, monocular cameras suffer from the lack of a third dimension for the detection of objects.

However, 3D object detection can be achieved by applying extrapolation of the detected 2D bounding boxes by reprojection constraints or regression models; nevertheless, the accuracy of depth calculations is low.

The stereo camera, on the other hand, a more expensive alternative, provides distance calculations but with higher computational requirements. Multiple monocular cameras have been used in [20] to achieve multi-object tracking. Additionally, in [21,22], various algorithms were developed to perform object detection and localization. So far, though, results have suffered from relatively low accuracy in depth estimation, especially at longer ranges.

#### 1.3.2. Point Clouds Acquired by LiDARs

LiDAR uses the Time of Flight (ToF) principle to detect the distance between the sensor and the detected objects. The maximum working detectable distance of LiDARs is 200 m [23]. LiDARs can withstand different weather and lighting conditions. Different LiDAR types project a different number of laser beams. Two-dimensional LiDARs project a single beam on a rotating mirror, while 3D LiDARs use multiple laser diodes that rotate at a very high speed; the higher the number of laser diodes, the more measurements can be acquired and the more accurate the perception task becomes [24]. Multiple 2D LiDARs (single beam) have been used in vehicle detection [25] and pedestrian detection [26,27] by applying pattern recognition techniques; however, this limits the detection to limited object classes.

Multi-beam LiDARs, on the other hand, are used for 3D object detection [28,29]. Examples of 2D LiDARs are: LRS-1000, LMS-291, and UTM-30LX, while examples of multi-beam LiDARs are IBEO LUX, Velodyne, and Quanergy. Three-dimensional LiDARs are more computationally extensive and suffer from higher costs.

There are three main methods for achieving 3D detection using point clouds [11]:

- Projection of a point cloud into a 2D plane in order to apply 2D detection frameworks to acquire 3D localization on projected images.
- Volumetric methods by voxelization [30,31]. However, 3D convolutional operations are computationally expensive.
- The use of PointNets [32,33,34] by applying raw point clouds directly to predict 3D bounding boxes. This method is also computationally expensive and increases running time.

#### 1.3.3. Sensor Fusion

Using a single type of sensor has proven to be insufficient and unreliable; sensor fusion is therefore mandatory in order to overcome these limitations. As a result of using multiple sensors, sensor fusion enhances the reliability and accuracy of measurements and reduces their uncertainty [5]. 

Many papers have applied sensor fusion to multi-beam LiDARs and cameras to achieve obstacle detection and avoidance. LiDAR was responsible for detecting the accurate position of objects, while the camera would detect its features and classification. Responding to the object detection problem, Han et al. [35] developed a framework that applied decision-level sensor fusion techniques on a Velodyne 64-beam LiDAR with RGB camera in order to improve the detection of dim objects such as pedestrians and cyclists. Additionally, a 3D object detector that processes in a Bird’s Eye View (BEV) is outlined in [36]; it fuses image features by learning to project them into the BEV space. Some approaches have targeted the detection of specific object classes: pedestrian pattern matching and recognition [37], vehicle detection [38], and passive beacon detection [39]. However, all of these papers either used expensive 3D LiDARs which acquire extensive amounts of data, or they suffered from limitations on the classes of detected objects. Table 1 lists the most popular and recent 3D object detection networks and frameworks along with their limitations.

Although LiDAR-based 3D detections have attracted many researchers, point clouds still lack the texture information that enables them to classify objects. Moreover, point clouds suffer from sparsity and decreased density when detecting distant objects. In this paper, therefore, a 2D LiDAR and a monocular camera are fused together in order to achieve real-time dynamic object detection for AVs. This research acts as a foundation for the employment of 2D LiDARs on AVs as a lower cost substitute for 3D LiDARs. It also addresses the challenge of the presence of multiple overlapping moving objects in the same scene with real-time constraints.

### 1.4. Paper Organization

The rest of this paper is organized as follows: Section 2 discusses the real-time object detection module using a monocular camera; Section 3 illustrates how the LiDAR measurements were processed; Section 4 explains the fusion methodology between the video-processing module and LiDAR measurements; Section 5 shows and discusses the results obtained from the work; finally, Section 6 presents the paper’s conclusion and discussion.

## 2. Real-Time Object Detection

### 2.1. Deep-Learning-Based Object Detection

Object detection can be defined as the process of detecting, localizing, and identifying the class of detected objects. Object detection methods output bounding boxes around detected objects, along with an associated predicted class and confidence score [19]. Different criteria affect the choice of the object detection algorithm, and, as a result, diverse driving scenarios impose different object detection challenges. For example:

- Variable weather and lighting conditions.
- Reflective objects.
- Diverse object sizes.
- The occlusion and truncation of obstacles.

In autonomous driving, objects that need to be detected are either static or dynamic. Traffic lights and signs, buildings, bridges, and curbs are considered static objects. Pedestrians, cyclists, animals, and vehicles, on the other hand, are considered dynamic objects due to their continuously varying locations and features. The detection of static objects is considered a straightforward task, which has been addressed in many previous studies (examples are shown in [45,46,47,48]). In this paper, therefore, we focus on the detection of vulnerable objects (e.g., dynamic objects) due to the greater levels of danger they pose during an AV’s driving process.

In this proposed research, a pre-trained deep-learning (DL)-based real-time object detection network, namely YOLOv3, is employed. YOLOv3 works upon Darknet, which is a neural network framework created by Joseph Redmon [49]. It is an open-source framework written by C/CUDA, and serves as the basis for YOLO. The original repository can be found in [50]. YOLOv3’s object detection network outputs 2D bounding boxes along with the classification of the detected objects. The model we used is pre-trained on KITTI [51,52], the largest computer vision evaluation dataset for autonomous driving scenarios in the world. It contains 7481 frames of training data and 7518 of test data. It has nine classes of labelled objects which we merged into six classes (Car, Van, Truck, Tram, Pedestrian, Cyclist).

### 2.2. Overlapping Detection

In order to use the 2D LiDAR accurately in diverse and challenging driving environments, and taking into consideration the presence of multiple different objects that could be overlapping and interacting with each other, an overlapping detection algorithm was necessary in order to detect which objects were overlapping with each other and forming clusters of objects. This algorithm will come into use during the LiDAR and camera integration step below.

In order to perform the overlapping detection, two attributes were added to each detected object: the first one holds the pixel ranges which are overlapping with other detected objects $P_{OL}$, and the second one holds the IDs of other objects that are sharing the pixels ($P_{OL}$) with the current object.

## 3. LiDAR Data Processing

### 3.1. Hokuyo UTM-30LX

**Figure 1:**
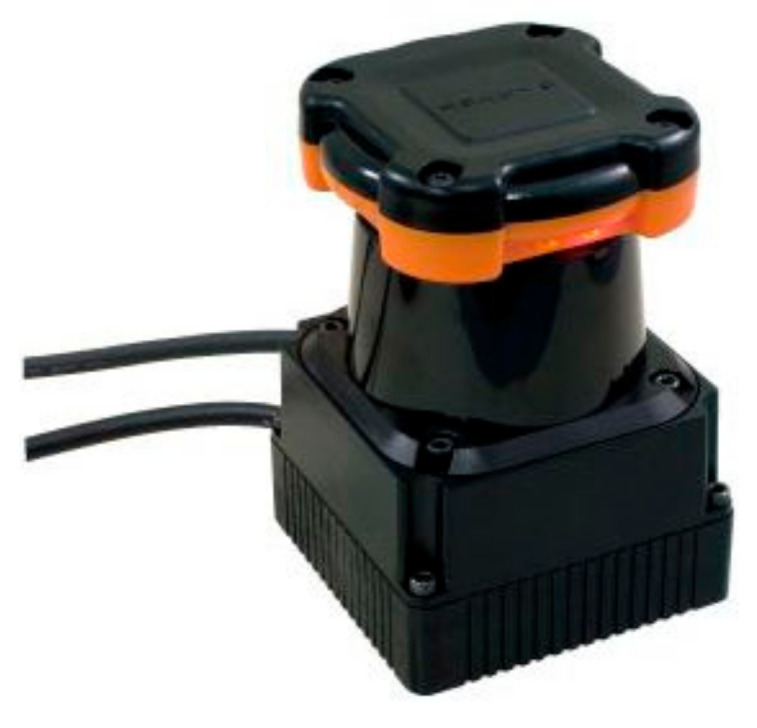
Hokuyo UTM 30LX.

The LiDAR used in this research is the Hokuyo UTM-30LX (shown in Figure 1). It is a 2D radial LiDAR that measures 1081 distance points in a range from −135° to 135°, where orientation 0° corresponds to the front of the LiDAR. The following represent its other specifications [53]:

- Range: 30 m.
- 270° scanning angle.
- 0.25° angular resolution.
- Light source: Laser semiconductor 870 nm, Laser class 1.
- Supply voltage: 12 VDC ± 10%.
- Supply current: a maximum of 1 A, normal is 0.7 A.
- Power consumption: less than 8 W.

### 3.2. Conversion of Radial Measurements into Perpendicular Measurements

**Figure 2:**
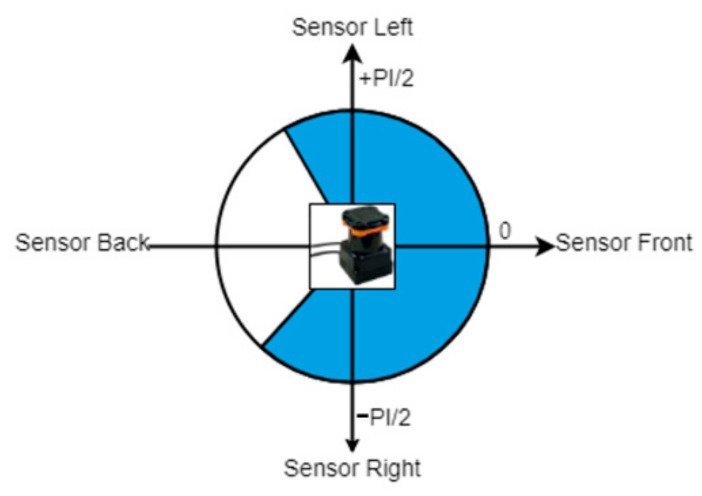
Hokuyo UTM 30LX angular range.

LiDAR works by measuring the distances in an angular rotational pattern; hence, the measurements acquired are radial, as shown in Figure 2. In order to normalize the LiDAR measurements, these radial measurements must therefore be converted into perpendicular measurements (as shown in Equation (1)).

⊥ distance = cos(Angle) × radial distance(1)

### 3.3. Linearization and Smoothing

Data acquired by the LiDAR for objects bounded by the bounding boxes cannot be considered a straightforward distance to be added as a third dimension of the detected objects. This is due to many factors; for example, the uneven surfaces of the detected objects, the sensors’ uncertainty, and the continuous overlapping and truncation of objects. Therefore, data acquired by the LiDAR are smoothed and linearized to give a better understanding of the surrounding objects.

LiDAR measurements are known to have a Gaussian noise distribution with a variance of ±3 cm. Therefore, a filter with a Gaussian impulse response function is a good candidate for noise suppression. Objects’ surfaces in real scenarios include both flat and rough surfaces or edges. Due to the sparsity of LiDAR measurements of distant objects, it is also assumed that the empty-depth pixels contain the same or similar measurements that also need to be restructured. Object edges are observed as a discontinuity in the LiDAR measurements, while flat surfaces have smoothly varying values. Thus, it is natural that the reconstruction of the depth measurements uses a form of edge-preserving filter. In order to maintain performance in real-time, a median filter was used to perform the filtering and preservation of edged features. 

### 3.4. Grouping of LiDAR Measurements into Clusters with Unique IDs

Different studies have addressed the problem of object segmentation on LiDAR point clouds [25,28,29,54,55]; however, they either used 3D point clouds or assumed that the world consists of separate objects that are not physically overlapping with each other. In this paper, we address the challenge of having multiple dynamic and interacting/overlapping objects.

After filtering LiDAR measurements, clustering is performed by grouping similar neighboring data readings and assigning a unique ID for each of them along with an average distance value. The two main variables in this step are:

- Minimum cluster size: in order to avoid the creation of numerous unneeded mini-clusters that may represent objects’ subregions, different cluster sizes were tested. The smaller the size of clusters, the more false clusters were created.
- Setting a threshold to the difference which sets the edge between consecutive clusters.

## 4. Camera and LiDAR Fusion

### 4.1. Sensor Placement

The vehicle’s ground clearance (i.e., ride height) must be considered during the placement of sensors: it is the shortest distance between a flat-level surface and the lowest part of a vehicle, other than those parts designed to be in contact with the ground such as tires and SIS. Eighty-two different vehicles (including SUVs) were surveyed in order to estimate the average ground clearance of vehicles in the UK so as to place the LiDAR at a height that was between the maximum ground clearance and the minimum vehicle height. It was concluded that the LiDAR’s optimal height was 559 mm away from the ground. In the proposed setup, the camera and the LiDAR should have a common horizontal center point.

### 4.2. Mapping between Image and LiDAR Coordinates 

The output from the video-processing module consists of:

- Two-dimensional bounding boxes drawn over image pixels.
- Object classes.

In order to apply complementary sensor fusion between pixels (bounding boxes) and LiDAR measurements, a mapping between image pixels and real-world angular coordinates is necessary. As we are using a 2D LiDAR, we are only concerned with the horizontal plane (x-axis), as the LiDAR has a constant vertical value.

**Figure 3:**
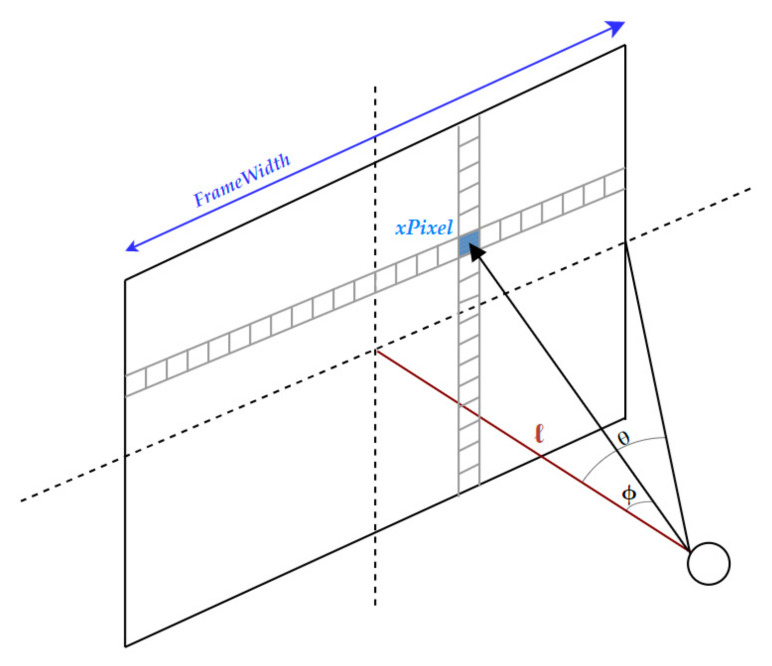
Conversion of pixel values into real-world angular coordinates.

Based on the camera pinhole mode shown in Figure 3, a function was developed in order to convert image pixels into angular rotations. Inputs to this function are:

- A pixel x-coordinate (xPixel).
- Width of the frame (FrameWidth).
- Horizontal field of view of the camera (HFOV).

Assuming a straight line is drawn between the camera and the center of the image (ℓ), two right-angled triangles can be drawn:

1. The hypotenuse goes from the camera to the edge of the image and has an angle (θ) formed between the hypotenuse and (ℓ).
2. The hypotenuse goes from the camera to (xPixel) and has an angle (ϕ) formed between its hypotenuse and (ℓ).

In this setup, the angle (ϕ) is required to be calculated. The following is the trigonometric calculation that is used to determine (ϕ):

b = FrameWidth/2(2)

θ = HFOV/2(3)

x = xPixel-Frame Center(4)

tan(θ) = b/ℓ(5)

tan(ϕ) = x/ℓ(6)

Making use of the common (ℓ) in both Equations (5) and (6), we solve both for (ℓ), namely:

ℓ = b/tan(θ) = x/tan(ϕ)(7)

Φ = tan^−1^ ((x tan(θ))/b) (8)

This process is performed on the left and right x-coordinates of each bounding box in order to convert the bounding boxes’ horizontal pixel values into real-world angular coordinates.

### 4.3. Complementary Camera and LiDAR Fusion

**Figure 4:**
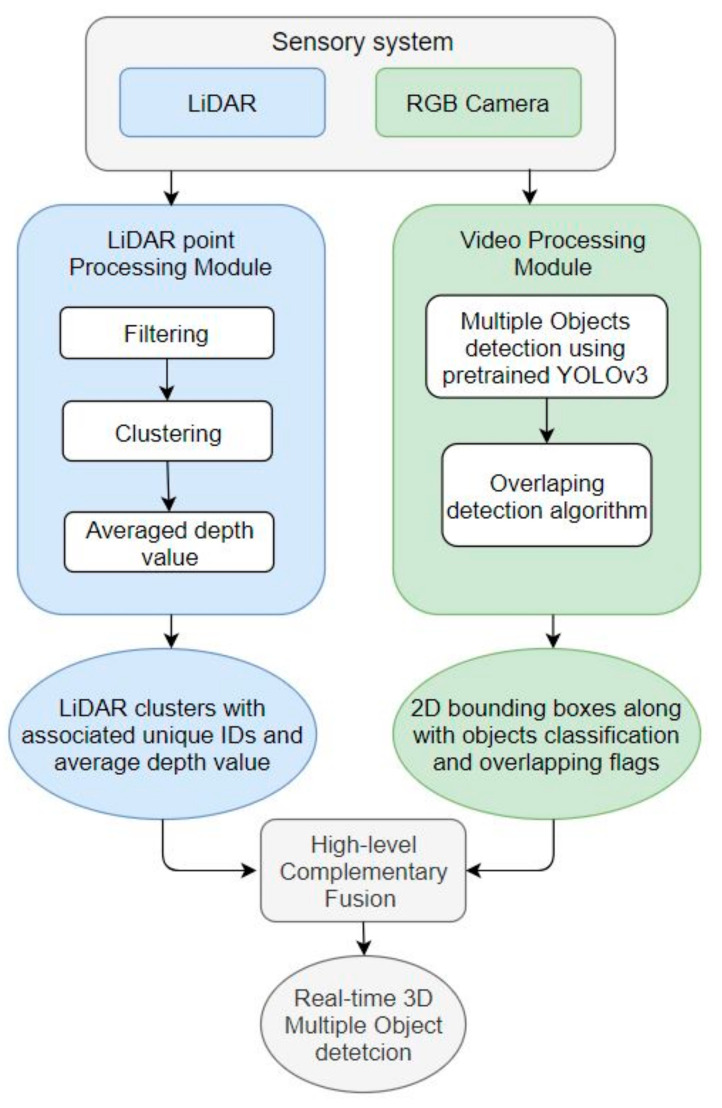
Block diagram for the system.

The direct fusion between the video and LiDAR data is a straightforward task that outputs a horizontal line of pixels with an associated distance measurement. However, the target of this process is to associate a depth measurement with the 2D bounding boxes. Therefore, when fusion is performed between bounding boxes and LiDAR measurements, the result is bounding boxes with an associated distance measurement. This task involves one main challenge: objects are normally overlapping with each other; therefore, the bounding boxes are not separate from each other, and pixels bounded by bounding boxes may include LiDAR measurements corresponding to multiple objects. Figure 4 is a block diagram that illustrates the fusion process between the video and LiDAR data-processing modules.

**Figure 5:**
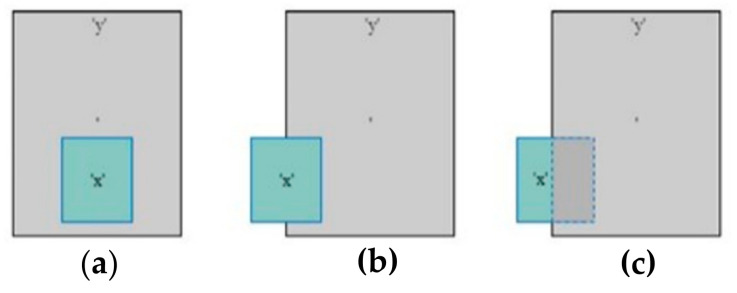
Different overlapping scenarios. (**a**) Object ‘x’ is fully in front of object ‘y’; (**b**) Object ‘x’ is partially in front of object ‘y’; (**c**) Object ‘x’ is partially behind object ‘y’.

In this step, sensor fusion is performed between the bounding boxes generated by the video-processing module and the clusters generated by the LiDAR data-processing module. There are multiple instances of overlapping between objects:

- No overlapping.
- Object ‘x’ is fully in front of object ‘y’ (object ‘x’ is smaller than object ‘y’) (as shown in Figure 5a).
- Object ‘x’ is partially in front of object ‘y’ (as shown in Figure 5b).
- Object ‘x’ is partially behind object ‘y’ (as shown in Figure 5c).

**Figure 6:**
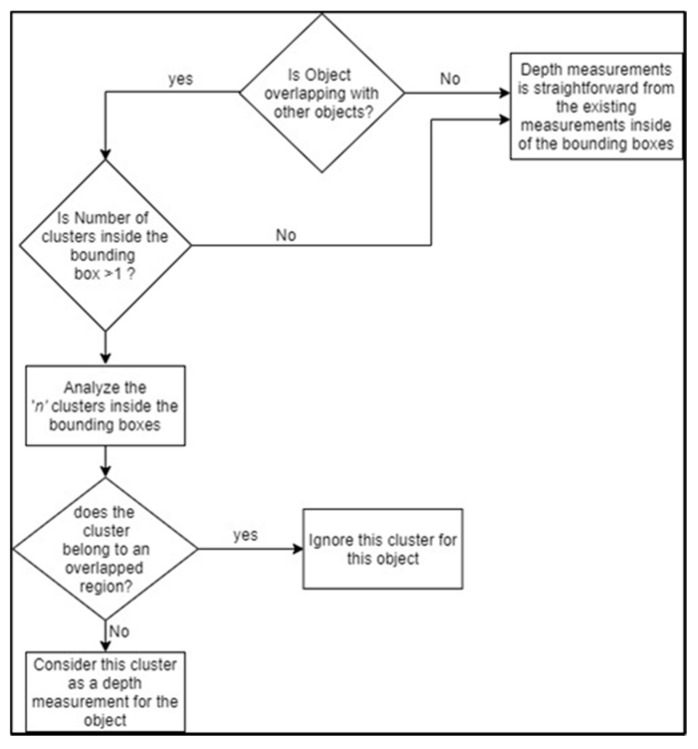
Flowchart for calculating the correct depth measurements for detected objects during overlapping objects.

In this operation, the algorithm analyzes the LiDAR clusters associated with the bounding boxes of each detected object. The flowchart for this operation is shown in Figure 6. This operation will complement the real-time object detection step made by the video-processing module because the bounding boxes are generally bigger than the true boundaries of the detected objects.

## 5. Results

### 5.1. Real-Time Object Detection

YOLOv3 has been chosen as the real-time object detector along with the KITTI dataset [51,52]. Our platform is configured with an Intel^®^ Core™ i7-8750H CPU and an NVIDIA GeForce GTX 1050Ti GPU, which is considered an average-performance GPU. When YOLOv3 is tested on the KITTI raw dataset, it achieves the results shown in Table 2. There are object detectors that perform better on the KITTI dataset (ex: Faster R-CCN); however, due to their slow execution speed, they cannot be used in real-time autonomous driving scenarios. Further comparisons between YOLOv3 and other deep learning object detection methods on different datasets are presented in [5,19].

### 5.2. Processing of LiDAR Measurements

**Figure 7:**
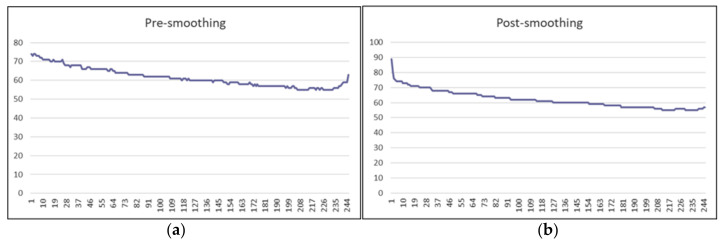
(**a**) A sample of LiDAR measurements on a rough surface pre-smoothing. (**b**) Same LiDAR measurements post-smoothing.

The first step in processing LiDAR measurements is performing median filtering in order to smooth the measurements while maintaining the edges. Figure 7a shows a sample of LiDAR measurements of rough surfaces before filtering, and Figure 7b shows the same measurements after filtering.

**Figure 8:**
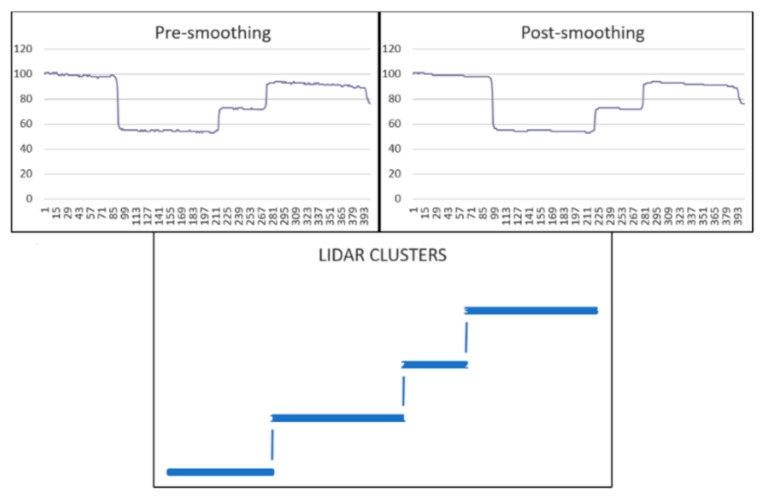
A sample of LiDAR measurements when scanning two overlapping objects.

The second step is dividing the LiDAR measurements into groups and assigning each group a unique ID. Figure 8 shows a sample of LiDAR measurements when two cars were present (one car is partially in front of the other). The output showed the detection of four clusters.

Due to the lack of 2D LiDAR point clouds, all the testing was performed in real-time driving scenarios, and the performance was manually measured and validated.

### 5.3. Adding a Third Dimension to Visual Bounding Boxes

The last step was making use of the LiDAR measurements (after filtering and grouping) in order to add a third dimension (depth) to the 2D bounding boxes generated from the real-time visual object detector. The system was tested in real-time scenarios, and it was capable of coping with the real-time constraints by performing in 18 FPS while maintaining dynamic object detection in addition to adding a depth dimension to the bounding boxes. The achieved running time of the proposed system is a major advancement compared to other approaches (refer to Table 1). The system was tested on moving vehicles, pedestrians, and cyclists in dynamic driving scenarios, while objects were overlapping and interacting with each other. However, a limitation of the system was the weather conditions, as the video-processing module is not robust enough for adverse weather conditions such as rain, snow, and fog.

## 6. Discussion

### 6.1. Conclusions

A monocular vision-based system is inexpensive and can achieve the required accuracy for obstacle detection in autonomous vehicles, but it only gives a 2D localization of objects. Therefore, a range-finder sensor should be used. However, 3D LiDARs are expensive and are hindering the widespread rollout of autonomous driving in both industry and research. In this study, a 2D LiDAR was adopted to develop a prototype for achieving reliable real-time multiple object detection in driving scenarios using lower cost sensors. 

### 6.2. Limitations and Future Work

The proposed research encourages the usage of low-cost 2D LiDARs in AVs, which advance the employment of autonomous driving in more vehicles. One limitation of the proposed method is that its performance is bound by the performance of the video-processing module (e.g., YOLOv3); therefore, further work should be conducted towards improving this, such as applying de-raining techniques. In terms of future work on the problem of multiple object detection based on the proposed research, the following approaches could be made:

- The use of multiple cameras in order to cover a wider horizontal field of view without causing much image distortion.
- Since the KITTI dataset only has daytime driving data, we suggest evaluating the real-time image-based object detection module on the Waymo Open Dataset.

## Figures and Tables

**Table 1:** Three-dimensional object detection networks and frameworks.

Paper	Modality	Limitation
Multi-task multi-sensor fusion for 3D object detection [40]	RGB + 3D point cloud	Expensive 3D LiDAR Not real-time
Frustum pointnets for 3D Object Detection from rgb-d data [34]	RGB-D	0.12 s per frame Not real-time
Pointfusion: deep sensor fusion for 3D bounding box estimation [41]	RGB + 3D point cloud	1.3 s per frame Not real-time
RoarNet: a robust 3D object detection based on regiOn approximation refinement [42]	RGB + 3D point cloud	Expensive 3D LiDAR Not real-time
A frustum-based probabilistic framework for 3D object detection by fusion of LiDAR and camera data [43]	RGB + 3D point cloud	Only for detecting static object
SEG-VoxelNet for 3D vehicle detection from RGB and LiDAR data [44]	RGB + 3D point cloud	Only detects vehicles Not real-time
MVX-Net: multimodal voxelnet for 3D object detection	RGB + 3D point cloud	Not real-time
3D-cvf: generating joint camera and lidar features using cross-view spatial feature fusion for 3D object detection	RGB + 3D point cloud	NVIDIA GTX 1080Ti, inference time 75 ms per frame (13.33 FPS)
PI-RCNN: an efficient multi-sensor 3D object detector with point-based attentive cont-conv fusion module	RGB + 3D point cloud	Not real-time
Image guidance-based 3D vehicle detection in traffic scene	RGB + 3D point cloud	Only vehicles, 4FPS
Epnet: enhancing point features with image semantics for 3D object detection.	RGB + 3D point cloud	Not real-time

**Table 2:** Mean Average Precision (MAP) of testing YOLOv3 on the KITTI dataset.

Benchmark	Easy	Moderate	Hard
Car	56%	36.23%	29.55%
Pedestrian	29.98%	22.84%	22.21%
Cyclist	9.09%	9.09%	9.09%

## Data Availability

Sharing not applicable. No new data were created or analyzed in this study. Data sharing is not applicable to this article.

## References

[1] Department for Transport (2015). The Pathway to Driverless Cars: Summary Report and Action Plan.

[2] Kaiwartya O., Abdullah A.H., Cao Y., Altameem A., Prasad M., Lin C., Liu X. (2016). Internet of vehicles: Motivation, layered architecture, network model, challenges, and future aspects. IEEE Access.

[3] Arena F., Pau G. (2019). An overview of vehicular communications. Future Internet.

[4] Ondruš J., Kolla E., Vertaľ P., Šarić Ž. (2020). How Do Autonomous Cars Work?. Transp. Res. Procedia.

[5] Khatab E., Onsy A., Varley M., Abouelfarag A. (2021). Vulnerable objects detection for autonomous driving: A review. Integration.

[6] Ahangar M.N., Ahmed Q.Z., Khan F.A., Hafeez M. (2021). A survey of autonomous vehicles: Enabling communication technologies and challenges. Sensors.

[7] Zhu H., Yuen K., Mihaylova L., Leung H. (2017). Overview of environment perception for intelligent vehicles. IEEE Trans. Intell. Transp. Syst..

[8] van Brummelen J., O’Brien M., Gruyer D., Najjaran H. (2018). Autonomous vehicle perception: The technology of today and tomorrow. Transp. Res. Part C Emerg. Technol..

[9] Yoneda K., Suganuma N., Yanase R., Aldibaja M. (2019). Automated driving recognition technologies for adverse weather conditions. IATSS Res..

[10] (2018). SAE On-Road Automated Vehicle Standards Committee and Others, Taxonomy and Definitions for Terms Related to Driving Automation Systems for On-Road Motor Vehicles.

[11] Dai D., Chen Z., Bao P., Wang J. (2021). A Review of 3D Object Detection for Autonomous Driving of Electric Vehicles. World Electr. Veh. J..

[12] Kovačić K., Ivanjko E., Gold H. (2013). Computer vision systems in road vehicles: A review. arXiv.

[13] Ilas C. Electronic sensing technologies for autonomous ground vehicles: A review. Proceedings of the 2013 8th International Symposium on Advanced Topics in Electrical Engineering (Atee).

[14] Aqel M.O., Marhaban M.H., Saripan M.I., Ismail N.B. (2016). Review of visual odometry: Types, approaches, challenges, and applications. SpringerPlus.

[15] Shi W., Alawieh M.B., Li X., Yu H. (2017). Algorithm and hardware implementation for visual perception system in autonomous vehicle: A survey. Integr. VLSI J..

[16] Campbell S., O’Mahony N., Krpalcova L., Riordan D., Walsh J., Murphy A., Ryan C. Sensor technology in autonomous vehicles: A review. Proceedings of the 2018 29th Irish Signals and Systems Conference (ISSC).

[17] Kocić J., Jovičić N., Drndarević V. Sensors and sensor fusion in autonomous vehicles. Proceedings of the 2018 26th Telecommunications Forum (TELFOR).

[18] Rosique F., Navarro P.J., Fernández C., Padilla A. (2019). A systematic review of perception system and simulators for autonomous vehicles research. Sensors.

[19] Haris M., Glowacz A. (2021). Road Object Detection: A Comparative Study of Deep Learning-Based Algorithms. Electronics.

[20] Yoon K., Song Y., Jeon M. (2018). Multiple hypothesis tracking algorithm for multi-target multi-camera tracking with disjoint views. IET Image Process..

[21] Mousavian A., Anguelov D., Flynn J., Kosecka J. 3d bounding box estimation using deep learning and geometry. Proceedings of the IEEE Conference on Computer Vision and Pattern Recognition.

[22] Chen X., Kundu K., Zhang Z., Ma H., Fidler S., Urtasun R. Monocular 3d object detection for autonomous driving. Proceedings of the IEEE Conference on Computer Vision and Pattern Recognition.

[23] Wang Z., Wu Y., Niu Q. (2019). Multi-sensor fusion in automated driving: A survey. IEEE Access.

[24] Asvadi, Garrote L., Premebida C., Peixoto P., Nunes U.J. (2018). Multimodal vehicle detection: Fusing 3D-LIDAR and color camera data. Pattern Recognit. Lett..

[25] Zhang X., Xu W., Dong C., Dolan J.M. Efficient L-shape fitting for vehicle detection using laser scanners. Proceedings of the 2017 IEEE Intelligent Vehicles Symposium (IV).

[26] Taipalus T., Ahtiainen J. Human detection and tracking with knee-high mobile 2D LIDAR. Proceedings of the 2011 IEEE International Conference on Robotics and Biomimetics.

[27] Shao X., Zhao H., Nakamura K., Katabira K., Shibasaki R., Nakagawa Y. Detection and tracking of multiple pedestrians by using laser range scanners. Proceedings of the 2007 IEEE/RSJ International Conference on Intelligent Robots and Systems.

[28] Rozsa Z., Sziranyi T. (2018). Obstacle prediction for automated guided vehicles based on point clouds measured by a tilted LIDAR sensor. IEEE Trans. Intell. Transp. Syst..

[29] García F., Jiménez F., Naranjo J.E., Zato J.G., Aparicio F., Armingol J.M., de la Escalera A. (2012). Environment perception based on LIDAR sensors for real road applications. Robotica.

[30] Shi S., Jiang L., Deng J., Wang Z., Guo C., Shi J., Wang X., Li H. (2021). PV-RCNN: Point-Voxel Feature Set Abstraction With Local Vector Representation for 3D Object Detection. arXiv.

[31] Zhou Y., Tuzel O. Voxelnet: End-to-end learning for point cloud based 3d object detection. Proceedings of the IEEE Conference on Computer Vision and Pattern Recognition.

[32] Yang Z., Sun Y., Liu S., Shen X., Jia J. Std: Sparse-to-dense 3d object detector for point cloud. Proceedings of the IEEE/CVF International Conference on Computer Vision.

[33] Qi C.R., Su H., Mo K., Guibas L.J. Pointnet: Deep learning on point sets for 3d classification and segmentation. Proceedings of the IEEE Conference on Computer Vision and Pattern Recognition.

[34] Qi C.R., Liu W., Wu C., Su H., Guibas L.J. Frustum pointnets for 3d object detection from rgb-d data. Proceedings of the IEEE Conference on Computer Vision and Pattern Recognition.

[35] Han J., Liao Y., Zhang J., Wang S., Li S. (2018). Target Fusion Detection of LiDAR and Camera Based on the Improved YOLO Algorithm. Mathematics.

[36] Liang M., Yang B., Wang S., Urtasun R. Deep continuous fusion for multi-sensor 3d object detection. Proceedings of the European Conference on Computer Vision (ECCV).

[37] García F., García J., Ponz A., de la Escalera A., Armingol J.M. (2014). Context aided pedestrian detection for danger estimation based on laser scanner and computer vision. Expert Syst. Appl..

[38] Garcia F., Martin D., de la Escalera A., Armingol J.M. (2017). Sensor fusion methodology for vehicle detection. IEEE Intell. Transp. Syst. Mag..

[39] Rövid A., Remeli V. Towards raw sensor fusion in 3D object detection. Proceedings of the 2019 IEEE 17th World Symposium on Applied Machine Intelligence and Informatics (SAMI).

[40] Liang M., Yang B., Chen Y., Hu R., Urtasun R. Multi-task multi-sensor fusion for 3d object detection. Proceedings of the IEEE/CVF Conference on Computer Vision and Pattern Recognition.

[41] Xu D., Anguelov D., Jain A. Pointfusion: Deep sensor fusion for 3d bounding box estimation. Proceedings of the IEEE Conference on Computer Vision and Pattern Recognition.

[42] Shin K., Kwon Y.P., Tomizuka M. Roarnet: A robust 3d object detection based on region approximation refinement. Proceedings of the 2019 IEEE Intelligent Vehicles Symposium (IV).

[43] Gong Z., Lin H., Zhang D., Luo Z., Zelek J., Chen Y., Nurunnabi A., Wang C., Li J. (2020). A Frustum-based probabilistic framework for 3D object detection by fusion of LiDAR and camera data. ISPRS J. Photogramm. Remote Sens..

[44] Dou J., Xue J., Fang J. SEG-VoxelNet for 3D vehicle detection from RGB and LiDAR data. Proceedings of the 2019 International Conference on Robotics and Automation (ICRA).

[45] Fernández C., Izquierdo R., Llorca D.F., Sotelo M.A. Road curb and lanes detection for autonomous driving on urban scenarios. Proceedings of the 17th International IEEE Conference on Intelligent Transportation Systems (ITSC).

[46] Vitas D., Tomic M., Burul M. (2020). Traffic Light Detection in Autonomous Driving Systems. IEEE Consum. Electron. Mag..

[47] Levinson J., Askeland J., Dolson J., Thrun S. Traffic light mapping, localization, and state detection for autonomous vehicles. Proceedings of the 2011 IEEE International Conference on Robotics and Automation.

[48] Mu G., Xinyu Z., Deyi L., Tianlei Z., Lifeng A. (2015). Traffic light detection and recognition for autonomous vehicles. J. China Univ. Posts Telecommun..

[49] Redmon J. (2013). Darknet: Open Source Neural Networks in C. https://pjreddie.com/darknet/.

[50] Darknet. https://github.com/pjreddie/darknet.

[51] Geiger A., Lenz P., Urtasun R. Are we ready for autonomous driving? The kitti vision benchmark suite. Proceedings of the 2012 IEEE Conference on Computer Vision and Pattern Recognition.

[52] Geiger A., Lenz P., Stiller C., Urtasun R. (2015). The KITTI Vision Benchmark Suite. Http://Www.Cvlibs.Net/Datasets/Kitti.

[53] Scanning Rangefinder Distance Data Output/UTM-30LX Product Details|HOKUYO AUTOMATIC CO., LTD. https://www.hokuyo-aut.jp/search/single.php?serial=169.

[54] Fang Z., Zhao S., Wen S., Zhang Y. (2018). A Real-Time 3D Perception and Reconstruction System Based on a 2D Laser Scanner. J. Sens..

[55] Choi D., Bok Y., Kim J., Shim I., Kweon I. (2017). Structure-From-Motion in 3D Space Using 2D Lidars. Sensors.

